# Influence of parental physical activity on offspring’s nutritional status: an intergenerational study in the 1993 Pelotas birth cohort

**DOI:** 10.1017/S1368980021004079

**Published:** 2022-08

**Authors:** Cauane Blumenberg, Rafaela Costa Martins, Shana Ginar da Silva, Bruna Gonçalves Cordeiro da Silva, Fernando C Wehrmeister, Helen Gonçalves, Pedro C Hallal, Inácio Crochemore-Silva, Ana MB Menezes

**Affiliations:** 1 Post-graduate Program in Epidemiology, Federal University of Pelotas (UFPel), 1160 Marechal Deodoro St., 3rd Floor, Centro, Pelotas, RS 96020-220, Brazil; 2 Grupo de Estudos e Pesquisa em Acelerometria (GEPEA), Pelotas, Brazil; 3 Medical School, Federal University of Fronteira Sul (UFFS), Passo Fundo, Brazil; 4 Post-graduate Program in Physical Education, Federal University of Pelotas (UFPel), Pelotas, Brazil

**Keywords:** Nutrition status, Physical activity, Public health, Intergenerational relations

## Abstract

**Objective:**

To investigate the influence of parental physical activity on offspring’s nutritional status in the 1993 Pelotas (Brazil) birth cohort.

**Design:**

Birth cohort study.

**Setting:**

The main outcomes were overweight and obesity status of children. The main exposure was parental physical activity over time, measured during the 11, 15 and 18 years of age follow-ups. The exposure was operationalised as cumulative, and the most recent measure before the birth of child. We adjusted Poisson regression models with robust variance to evaluate crude and adjusted associations between parental physical activity and offspring’s nutritional status. All analyses were stratified according to the sex of the parent.

**Participants:**

A total of 874 members from the 1993 Pelotas (Brazil) birth cohort followed-up at 22 years of age with their first-born child were analysed.

**Results:**

Children were, on average, 3·1 years old. Crude analyses showed that the mother’s cumulative physical activity measure had an indirect association with the prevalence of children’s obesity. The most recent maternal physical activity measure before the birth of the child was associated with 41 % lower prevalence of obesity in children, even after adjustment for confounders.

**Conclusions:**

The most recent maternal physical activity measure was indirectly associated with the prevalence of obesity in children. No associations were found for fathers, reinforcing the hypothesis of a biological effect of maternal physical activity on offspring’s nutritional status.

In 1953, the study by Morris and colleagues revealed that physical activity could prevent coronary heart diseases^(1)^. As the time passed by, the benefits of physical activity were found to be even more important, promoting well-being and preventing not only coronary heart diseases, but also other non-communicable diseases. Current knowledge links physical activity to other health benefits, such as preventing all-cause mortality, several types of cancer, type 2 diabetes, mental illnesses, hypertension and chronic obstructive pulmonary disease^(2–4)^. Besides, physically active individuals have a higher life expectancy compared to inactive individuals^(5)^.

In the 1990s, David Barker proposed the foetal origins hypothesis, which suggests that mother’s behaviours during pregnancy can impact future outcomes on their children^(6)^. This hypothesis brought attention to the role of intergenerational effects, also investigating the role of the father and other periods of parental life course, including those previous to the pregnancy^(7–10)^. Some studies showed that children born to obese or hyperglycaemic mothers are more likely to develop diabetes or obesity later in life^(7,11,12)^, that parents’ alcohol consumption influences adolescent’s drinking behaviour^(8)^, and that parental drug use predicts adolescent’s drug use^(9)^. However, few studies investigated the intergenerational effects of parental physical activity on offspring’s health-related indicators^(13)^, especially regarding their nutritional status.

The limited evidence about the intergenerational effects of physical activity on nutritional status is mixed. A study showed that reduced maternal physical activity over time was associated with higher offspring’s BMI^(10)^, while another showed no influence^(14)^. In order to further explore this subject, the members of the 1993 Pelotas birth cohort and their children were assessed to investigate the influence of parental physical activity measured in three moments previous to the birth of their child on the offspring’s nutritional status.

## Methods

### Sample

This study is inserted in the context of the 1993 Pelotas birth cohort. Between January and December 1993, all mothers living in the urban areas of Pelotas (Southern Brazil) and who gave birth to one or more liveborn children in maternity hospitals of the city were invited to enrol their children into the 1993 Pelotas birth cohort. A total of 5249 children born in 1993 (99·7 % of all births occurring in the city) agreed to participate in the longitudinal study^(15)^. Apart from the perinatal study, that occurred in 1993, the members of the birth cohort are routinely accompanied, and 13 follow-ups have been carried out so far. In 2015, as part of the 22 years of age follow-up of the 1993 birth cohort, the research team tried to locate through phone calls, social networks and official registries all its 5249 members, of which 3810 were interviewed and examined.

When invited to participate, the members of the birth cohort were also asked if they had any children. In case of an affirmative answer, they were asked to bring their children along to participate. A total of 948 members of the birth cohort (24·9 % of those followed-up at 22 years of age) attended the research clinic with their children, totalising 1213 children assessed (mean of 1·3 children per member of the birth cohort) – however, in this study only the first-born child is considered to simplify analyses and interpretation (*n* 948). This was the first follow-up of the ‘second generation’ of the 1993 Pelotas birth cohort. The second generation was assessed from January to December 2016, being composed of children born from mothers or fathers that are original members of the 1993 Pelotas birth cohort. Further details about the 22 years of age follow-up and the second generation sample can be found elsewhere^(16)^.

### Outcomes

BMI-for-age of the second generation was the main outcome analysed. Using children’s sex, age, height and weight, the Anthro Plus Stata macro was used to estimate the anthropometric index based on the 2007 WHO reference population^(17)^. Children’s age was calculated in months by subtracting the date that the children attended the research clinic from their date of birth. The height for children younger than 3 years was measured in centimetres with 0·1 cm precision with children lying down on an infant anthropometer. Children older than 3 years were measured standing on a stadiometer with 0·1 cm precision. The weight was assessed in kilograms using a regular scale with 0·1 kg precision. Children younger than 3 years were weighted with their mothers or fathers, followed by a weighting of the mother or father alone. A simple subtraction between both weights was performed to achieve children’s weight. In turn, children older than 3 years were weighted directly on the scale. All parents and children were weighted in very light clothing.

Outcomes were analysed as binary variables (overweight and obesity). Overweight was defined as children with *Z*-scores of BMI-for-age higher than +1 SD, and obesity was defined as children with *Z*-score of BMI-for-age higher than +2 SDs^(17,18)^.

### Exposures

Exposures analysed were based on parental physical activity. Total time on physical activity was investigated when the parents (original members of the 1993 Pelotas birth cohort) were 11, 15 and 18 years of age. In order to investigate how many minutes individuals spent on physical activity during a regular week, a list of physical activities practiced was applied in the 11 and 15 years follow-ups. For the 18 years follow-up, the transport and leisure time section of the International Physical Activity Questionnaire (IPAQ) was applied^(15,16,19)^. Both questionnaires investigate the number of days and the length of practice (in minutes) of each activity in the week before the interview. The number of minutes was multiplied by the number of days that each activity was practiced in the previous week to generate the minutes of physical activity in a regular week. Since different physical activity intensities were combined into a single measure, minutes on vigorous physical activities were weighted and further multiplied by two, following recommendations^(20,21)^. The intensity of each activity from the list used in the 11- and 15-year follow-ups was determined based on metabolic equivalents. Activities with metabolic equivalents higher than six were considered vigorous^(22)^. In contrast, the IPAQ already has established categories of physical activities according to different intensities^(19)^.

Two operationalisations of the exposure were analysed: a cumulative measure and the most recent physical activity measure before the birth of the child. The cumulative measure is the sum of follow-ups (0, 1, 2 or 3) previous to the birth of the second generation in which the parents were considered physically active. For instance, if the second generation was born when the parent was 16 years of age, only the 11- and 15-year follow-ups were considered in the sum.

For the most recent physical activity measure before the birth of the child, only the status (active or inactive) of the parent in the follow-up immediately before the birth of the second generation was considered. A total of 159 parents did not attend the follow-up immediately before the birth and were not considered in the analyses based on the most recent physical activity measure before the birth of the second generation. For all exposures, the parent had to spend at least 300 min/week in physical activity in the 11- and 15-year follow-ups, and at least 150 min/week in the 18-year follow-up to be considered active, according to WHO’s recommendations^(23)^.

### Statistical analyses

Crude and adjusted analyses were performed to assess the influence of exposures (father’s and mother’s physical activity) on the outcomes (offspring’s nutritional status). BMI-for-age when parents were 11 years of age, and the age of the child from the second generation were considered as confounders (all inserted in adjusted models as continuous variables). The parents’ BMI-for-age at 11 years of age was estimated using the same methodology applied for the second generation. We did not adjust for parental BMI at age 15 or 18 as these measures would lie within the pathway between exposures and outcomes, being mediators and not confounders of the associations. All analyses were stratified according to the sex of the parent.

Poisson regression models with robust variance were fitted to assess the influence of each parental physical activity exposure on second generation’s overweight and obesity status (binary outcomes). For binary outcomes, it is possible to interpret incidence rate ratios produced by Poisson regressions as prevalence ratios – as described by Barros and Hirakata^(24)^.

Our units of analysis were the second generation children, and when the parent had more than one child, we considered only the first born for each parent in order to simplify analyses and interpretation. All fathers and mothers in the sample had the same age (all born in 1993), however the number of follow-ups considered in the operationalisation of the exposures could vary. This could happen since only the physical activity measures previous to the birth of the second generation were considered in the operationalisation of exposures. Hence, the number of follow-ups considered for each parent depended on the age that the parent was when the first child was born. To correct for the different lengths of exposure (represented here by the number of follow-ups), the natural logarithm of the parental age at the moment the first child was born (age from conception) was added as an offset to Poisson regression models.

All analyses were performed using Stata software version 16.1 (StataCorp., LLC).

## Results

Out of the 3810 members of the 1993 Pelotas birth cohort interviewed during the 22 years of age follow-up, 948 attended the research clinic with their children. However, we analysed 874 parent-child pairs – since only those with complete information on the outcomes were considered (*n* 74 excluded due to missing information). A flowchart presenting the full sample and subsamples analysed is presented in Supplemental Fig. 1. Compared to the sample interviewed when they were 22 years old (*n* 3810), parents of the second generation (*n* 874) were poorer and mainly represented by females (73·5 % in analysed sample and 53·2 % in interviewed sample). Median minutes in physical activity were similar for the 11- and 15-year measures, but almost 50 % higher at 18 years of age for the full sample compared to the analysed. Mean BMI values were similar between the compared samples (see online Supplemental Table 1). The characteristics of the second generation samples analysed (*n* 874) and not included in analyses due to missing information on the outcome (*n* 74) are compared in Supplemental Table 2. Second generation’s children not included in the analyses were older and more frequently of male sex compared to the analysed sample. The median parental physical activity levels were similar.

The second generation was, on average, 3·1 years old (sd = 2·1) and mainly female (Table 1). Slightly more than one-third of the second generation presented overweight and 12·4 % was obese. Most of the 1993 Pelotas birth cohort members analysed were female (73·5 %). The median minutes of physical activity practice were similar in the 11- and 18-year follow-ups (approximately 290 min), and around 30 min lower in the 15-year follow-up. Almost 35 % of the parents were active only in one follow-up, and 70 % were considered active in the follow-up immediately before the birth of the second generation.

**Table 1:** Second generation’s and parent’s characteristics of the analysed sample

Second generation’s characteristics (*n* 874)	Mean		95 % CI
Age (years)	3·1		2·96, 3·24
BMI-for-age (*Z*-score)	0·75		0·66, 0·84
Sex	*n*	%	95 % CI
**Female	473	54·1	50·8, 57·4
**Male	401	45·9	42·6, 49·2
Overweight	317	36·3	33·2, 39·6
Obesity	108	12·4	10·3, 14·7
Parent’s characteristics (*n* 874)			
Physical activity (minutes)	Median		IQR
**11 years follow-up	290		140, 555
**15 years follow-up	265		125, 540
**18 years follow-up	292·5		120, 725
Sex	*n*	%	95 % CI
**Female	642	73·5	70·4, 76·3
**Male	232	26·5	23·7, 29·6
Physically active follow-ups			
**0	172	19·7	17·1, 22·5
**1	305	34·9	31·8, 38·1
**2	273	31·2	28·2, 34·4
**3	124	14·2	12·0, 16·7
Most recent physical activity status before the birth of the child			
**Inactive	212	29·6	26·4, 33·1
**Active	503	70·4	66·9, 73·6

IQR, interquartile range.

The cumulative physical activity measure, analysed as the number of physically active follow-ups, had no influence on the second generation’s prevalence of overweight (Table 2). In turn, crude analyses showed that the higher the number of active follow-ups in which mothers were active, the lower was the prevalence of obesity for the second generation. However, the association did not persist after adjustment for parental BMI-for-age at 11 years and child’s age. No association between cumulative physical activity and obesity was found for fathers, either in crude or adjusted analyses.

**Table 2:** Crude and adjusted prevalence ratio for overweight and obesity according to the number of physically active follow-ups by the parents (cumulative physical activity measure)

	Overweight				Obesity			
	Crude PR	95 % CI	Adjusted PR	95 % CI*	Crude PR	95 % CI	Adjusted PR	95 % CI*
Father’s active follow-ups	*P* = 0·322		*P* = 0·117		*P* = 0·323		*P* = 0·512	
**0	1·00		1·00		1·00		1·00	
**1	1·70	0·60, 4·79	2·67	0·85, 8·34	2·18	0·30, 15·75	3·09	0·49, 19·45
**2	1·30	0·46, 3·64	2·02	0·66, 6·21	1·61	0·23, 11·42	2·48	0·42, 14·43
**3	1·79	0·65, 4·96	2·91	0·94, 9·00	0·84	0·11, 6·63	1·65	0·24, 11·21
Mother’s active follow-ups	*P* = 0·177		*P* = 0·161		*P* = 0·014		*P* = 0·069	
**0	1·00		1·00		1·00		1·00	
**1	0·81	0·62, 1·04	0·83	0·64, 1·06	0·55	0·34, 0·89	0·61	0·38, 0·97
**2	0·74	0·56, 0·98	0·75	0·57, 1·00	0·49	0·28, 0·84	0·54	0·32, 0·92
**3	0·90	0·62, 1·31	1·00	0·68, 1·47	0·37	0·14, 0·99	0·50	0·19, 1·34

PR, prevalence ratio.

* Adjusted by father’s and mother’s BMI-for-age at 11 years of age and age of the child.

The effect of the most recent physical activity measure before the birth of the child, which only considered the follow-up immediately before the birth of the second generation, was also analysed (Table 3). No associations between paternal nor maternal physical activity and second generation overweight were found. In crude analyses, the prevalence of obesity in children born to active mothers was 41 % lower (prevalence ratio = 0·59; (95 % CI 0·39, 0·89)) compared to those born to inactive mothers. The association persisted even after adjustment for confounders, being the prevalence of obesity in children born to active mothers also 41 % lower (prevalence ratio = 0·59; (95 % CI 0·39, 0·87)) compared to their peers. In turn, there was no association between the most recent physical activity measure before the birth of the child and obesity of the second generation when fathers were analysed.

**Table 3:** Crude and adjusted prevalence ratio for overweight and obesity according to parental physical activity status in the most recent measure before the birth of the second generation

	Overweight				Obesity			
	Crude PR	95 % CI	Adjusted PR	95 % CI*	Crude PR	95 % CI	Adjusted PR	95 % CI*
Father’s physical activity status	*P* = 0·569		*P* = 0·372		*P* = 0·871		*P* = 0·878	
**Inactive	1·00		1·00		1·00		1·00	
**Active	1·15	0·71, 1·87	1·24	0·78, 1·97	0·93	0·37, 2·31	1·07	0·45, 2·56
Mother’s physical activity status	*P* = 0·246		*P* = 0·404		*P* = 0·012		*P* = 0·008	
**Inactive	1·00		1·00		1·00		1·00	
**Active	0·92	0·74, 1·15	0·91	0·74, 1·13	0·59	0·39, 0·89	0·59	0·39, 0·87

Analyses for this exposure consider only parents that attended to the most recent follow-up before the birth or the second generation (*n* 715).

PR, prevalence ratio.

* Adjusted by father’s and mother’s BMI-for-age at 11 years of age and age of the child.

## Discussion

This study examined the influence of parental physical activity on offspring’s nutritional status. Our results showed that the effect of the most recent maternal physical activity measure before the birth of the child was relevant to reduce the prevalence of obesity among children born to active mothers. In contrast, no associations between cumulative, or the most recent physical activity measure before the birth of the second generation and offspring’s nutritional status were found when fathers were analysed.

It is well-documented that genetic factors influence the offspring’s nutritional status^(25–27)^. However, shared environmental factors also play a substantial role regarding offspring’s overweight/obesity^(10,25,26,28,29)^. Nonetheless, few studies have focused on the influence of parental physical activity over time on offspring’s nutritional status, especially in population-based samples. The evidence available does not show consistent results. In a previous study, conducted in Norway with over 4000 parent-offspring dyads, a decrease in maternal physical activity levels was associated with a 0·1 increase on BMI *Z*-scores in adolescents. Father’s lifestyle changes, however, did not significantly affect adolescent’s BMI^(10)^. In the HUNT study, which included 3681 adolescents (15·9 years of age), Fasting and co-authors (2011) did not find any direct association between low levels of parental physical activity and offspring’s overweight^(30)^. In a population-based birth cohort from the Netherlands with 1554 children, Sijtsma *et al.* (2015) revealed no correlation between total, light, moderate or vigorous physical activity of the mother or father, assessed in one time point, with the children’s BMI or waist circumference *Z*-scores (3·9 years of age). However, the same study demonstrated that more active commuting by the mother was related to lower offspring’s BMI^(14)^. This last finding is in line with our results on the association between active mothers in the most recent measure before the birth of the child and reduced offspring’s obesity prevalence.

Intergenerational transmission, which is the transfer of individual traits, abilities and behaviours from parents to their children, is described to be one of the factors that can impact children’s outcomes and health behaviour^(31)^. Previous studies found strong correlations between a large range of parents’ and offspring’s outcomes, such as self-assessed health^(32)^, obesity and anthropometric measures^(33,34)^, mental health^(35)^, chronic health conditions^(36)^ and health-related behaviours^(8,37–40)^. For example, parental smoking experience is significantly associated with their offspring’s initiation and lifetime smoking^(38,39)^. Also, it is well established that BMI and obesity are strongly correlated among biological parent–child dyads^(34)^. However, in terms of the influence of parents’ physical activity over time on offspring’s nutritional status, the intergenerational transmission is not completely understood and lacks supportive evidence. Our findings showed an indirect association between the most recent maternal physical activity measure and children’s obesity prevalence. However, we found no association between paternal physical activity and offspring’s nutritional status. Thus, we hypothesise that biological factors might play a stronger role compared to environmental factors in the relationship between parental physical activity and offspring’s nutritional status.

To the best of our knowledge, there are no reports addressing the biological mechanisms involved in the potential intergenerational effects of the parents’ physical activity on their children’s growth. One hypothesis that might explain this influence is the theory of foetal programming, which indicates that the environment surrounding the foetus during its development, including maternal behaviours during pregnancy, plays a seminal role in determining its disease risk and health behaviours during the life^(6)^. An experimental examination demonstrated that a higher level of physical activity during pregnancy was related to a lower percentage of body fat at age five^(41)^. Also, the literature has shown that mothers who perform physical activity before and during pregnancy are less likely to have obese children in childhood and adulthood^(42,43)^. We found that the prevalence of obesity was lower in children born to mothers who were active in the follow-up immediately before the child’s birth. Our hypothesis for this finding is that mothers who were active in the follow-up prior to the child’s birth would be more likely to be active also during pregnancy. In our study, we did not have information regarding the measures of physical activity during pregnancy. However, the literature has shown that one of the strongest determinants of physical activity during pregnancy is the pre-pregnancy physical activity status^(44)^.

Parental lifestyle has a strong effect on children’s health, playing an important role in their habits, including healthy diet and physical activity^(45,46)^. This was also why we expected to find an association between parental physical activity and children’s nutritional status. However, it is known that child nutritional status is multifactorial and complex, driven by an interaction between genetic, biological and environmental factors^(47–49)^. Perinatal factors, birth size, catch-up growth, breastfeeding status, eating behaviours and physical activity are some of the factors affecting child nutritional status^(47)^. Parental BMI and height are also associated with offspring’s BMI and height^(48,50,51)^. Although it is important to understand the complete mechanism between parental physical activity and offspring’s nutritional status, our study is limited to understand the total effect of this association. Our findings are an important starting point to guide future studies specifically focused on understanding the direct, indirect and total effects of this association.

Our study has important strengths. It was carried out in a population-based birth cohort with high rates of follow-up, minimizing the likelihood of selection bias. The weight and height were assessed by trained technicians, providing high-quality measures. Parental physical activity was explored from different perspectives, which allowed us to evaluate the cumulative, and the effects of the most recent father’s and mother’s physical activity measure before the birth of the child on the offspring’s nutritional status.

Some limitations also have to be mentioned. Self-reported data and the different questionnaires used to assess parental physical activity might have overestimated the amount of physical activity. A second limitation is that we were not able to estimate the direct or indirect effects of parental physical activity on offspring’s nutritional status, but only the total effect. This happens because some perinatal mediators, such as offspring’s diet, physical activity and genetic factors were not available in our study. Also, we did not have information on maternal physical activity during pregnancy – which may have a specific effect on offspring’s body composition. Our analyses were adjusted by the parental BMI-for-age when they were 11 years old, at the same moment the first physical activity assessment was performed. Thus, we are not able to rule out the possibility that the parental physical activity influences the BMI-for-age at 11 years of age. The second generation includes participants that were evaluated at different ages. We performed the analyses with nutritional status variables standardised for age in order to minimise this difference. However, we can speculate that the effect of parental physical activity on offspring’s nutritional status may be different in early and late childhood – but we could not assess this due to the limited sample size. Additionally, there is some evidence that adolescent pregnancy may affect foetal growth^(52,53)^. However, in our study, only one-third of female participants were younger than 18 years old when became pregnant. Still, our results should be interpreted in light of the characteristics of the sample included in this study – young adults of up to 22 years of age. Finally, performing complete case analyses reflected on excluding around 8 % (*n* 74) of the children due to missing information. This could have affected the statistical power of our study by reducing the sample size and influenced the representativeness of the sample included in our analyses – since the frequency of male children and their mean age were higher in non-analysed children compared to our sample.

## Conclusion

Our results showed that the most recent physical activity measure before the birth of the child was associated with lower prevalence of obesity among children born to active mothers. Although without statistical significance, the findings showed a consistent direction of the effects for cumulative physical activity measures – where children born to active mothers had, in general, lower prevalence of overweight and obesity. In contrast, fathers’ physical activity did not influence offspring’s nutritional status. The fact we found association only for mothers, reinforce the hypothesis of a possible biological effect of maternal physical activity on the offspring’s nutritional status. Further studies, comprising increased sample sizes and exploring the mediators involved in the intergenerational effect of parent’s physical activity on their children’s growth are encouraged to provide a better understanding of the mechanisms of this association.

## Supplementary material

For supplementary material accompanying this paper visit https://doi.org/10.1017/S1368980021004079. click here to view supplementary material
