# Supplementary material for: Influence of parental physical activity on offspring’s nutritional status: an intergenerational study in the 1993 Pelotas birth cohort
Source: Public Health Nutr. 2021 Sep 27;25(8):2206–13. doi: 10.1017/S1368980021004079 (PMC9991797; doi:10.1017/S1368980021004079)
Supplement: Supplementary file 1 [file S1368980021004079sup.zip › S1368980021004079sup001.docx]

Supplementary table 2. Comparison of second generation’s and parental characteristics between the analysed sample and those not included in analyses.

| **Second generation's characteristics** | | |  |  |
| --- | --- | --- | --- | --- |
|  | **Analysed sample**  **(n = 874)** | | **Sample not included in analyses (n = 74)^a^** | |
|  | **Mean** | **95% CI** | **Mean** | **95% CI** |
| **Age (years)** | 3.1 | 2.96; 3.24 | 4.5 | 3.96; 5.00 |
| **BMI-for-age (Z-score)** | 0.75 | 0.66; 0.84 | - ^b^ | - ^b^ |
| **Sex** | **N (%)** | **95% CI** | **N (%)** | **95% CI** |
| Female | 473 (54.1) | 50.8; 57.4 | 36 (48.7) | 37.3; 60.1 |
| Male | 401 (45.9) | 42.6; 49.2 | 38 (51.3) | 40.0; 62.7 |
| **Overweight** | 317 (36.3) | 33.2; 39.6 | - ^b^ | - ^b^ |
| **Obesity** | 108 (12.4) | 10.3; 14.7 | - ^b^ | - ^b^ |
| **Parent's characteristics** | | |  |  |
|  | **Analysed sample**  **(n = 874)** | | **Sample not included in analyses (n = 74)^a^** | |
| **Physical activity (minutes)** | **Median** | **IQR** | **Median** | **IQR** |
| 11 years follow-up | 290 | 140; 555 | 270 | 134; 440 |
| 15 years follow-up | 265 | 125; 540 | 270 | 140; 530 |
| 18 years follow-up | 292.5 | 120; 725 | 210 | 80; 420 |
| **Sex** | **N (%)** | **95% CI** | **N (%)** | **95% CI** |
| Female | 642 (73.5) | 70.4; 76.3 | 63 (85.1) | 74.9; 91.7 |
| Male | 232 (26.5) | 23.7; 29.6 | 11 (14.9) | 8.3; 25.1 |
| CI, confidence interval. IQR, interquartile range.  ^a^ Parent-child pairs interviewed at the 22 years of age follow-up, but not included in the analyses due to missing information on the outcome (BMI-for-age for the second generation).  ^b^ Information not available. | | | | |
