# Supplementary material for: Influence of parental physical activity on offspring’s nutritional status: an intergenerational study in the 1993 Pelotas birth cohort
Source: Public Health Nutr. 2021 Sep 27;25(8):2206–13. doi: 10.1017/S1368980021004079 (PMC9991797; doi:10.1017/S1368980021004079)
Supplement: Supplementary file 1 [file S1368980021004079sup.zip › S1368980021004079sup002.docx]

**Full sample:**

3,810 members of the cohort interviewed at the 22 years of age follow-up

**Second generation sample:**

948 members of the cohort that attended to the research clinic with their children

**Analysed sample (complete cases):**

874 members of the cohort and their first-born child with complete information

74 members of the cohort and their first-born child excluded due to missing information on the outcome

2,862 members of the cohort that had no children or that did not attend to the research clinic with their children

Supplementary figure 1. Flowchart describing the samples and subsamples analysed in the study.
