# Supplementary material for: Influence of parental physical activity on offspring’s nutritional status: an intergenerational study in the 1993 Pelotas birth cohort
Source: Public Health Nutr. 2021 Sep 27;25(8):2206–13. doi: 10.1017/S1368980021004079 (PMC9991797; doi:10.1017/S1368980021004079)
Supplement: Supplementary file 1 [file S1368980021004079sup.zip › S1368980021004079sup003.docx]

Supplementary table 1. Comparison of parental characteristics between the analysed sample and those interviewed during the 22 years of age follow-up.

| **Parent's characteristics** | | |  |  |
| --- | --- | --- | --- | --- |
|  | **Analysed sample**  **(n = 874)** | | **Sample interviewed at the 22 years of age follow-up**  **(n = 3,810)** | |
| **Physical activity (minutes)** | **Median** | **IQR** | **Median** | **IQR** |
| 11 years follow-up | 290 | 140; 555 | 280 | 140; 540 |
| 15 years follow-up | 265 | 125; 540 | 280 | 120; 570 |
| 18 years follow-up | 292.5 | 120; 725 | 420 | 160; 870 |
| **Family income (BRL)^a^** | 1,720 | 1,065; 2,510 | 2,400 | 1,400; 3,700 |
|  | **Mean** | **95% CI** | **Mean** | **95% CI** |
| **BMI (kg/m^2^)^a^** | 26.2 | 25.8; 26.6 | 25.2 | 25.1; 25.4 |
| **Sex** | **N (%)** | **95% CI** | **N (%)** | **95% CI** |
| Female | 642 (73.5) | 70.4; 76.3 | 2,027 (53.2) | 51.6; 54.8 |
| Male | 232 (26.5) | 23.7; 29.6 | 1,783 (46.8) | 45.2; 48.4 |
| BMI, body mass index. BRL, Brazilian *Reais*. CI, confidence interval. IQR, interquartile range.  ^a^ Information collected during the 22 years of age follow-up | | | | |
